# Glia and the Synapse: Plasticity and Disease

**DOI:** 10.1155/2014/246714

**Published:** 2014-03-18

**Authors:** Irina Nikonenko, Emma Victoria Jones, Hubert Fiumelli, Yann Bernardinelli

**Affiliations:** ^1^Department of Neuroscience, School of Medicine, University of Geneva, 1211-CH Geneva, Switzerland; ^2^Centre for Research in Neuroscience, Department of Neurology and Neurosurgery, The Research Institute of the McGill University Health Centre, Montreal General Hospital, Montreal, QC, Canada H3G 1A4; ^3^Biological and Environmental Sciences & Engineering Division, King Abdullah University of Science and Technology, Thuwal 23955, Saudi Arabia

Glial cells play multiple, diverse roles in the central nervous system (CNS), ranging from the basal support of neuronal function to close partnership with the synapse. Growing experimental evidence shows the importance of glia for proper brain functioning and their involvement in injury and disease. Astrocytes are the most intriguing cells among the glial family. It is well known that they provide energetic substrates to neurons, take up neurotransmitters, and maintain ion homeostasis. Recent research has revealed that they can also release gliotransmitters and signaling molecules as well as maintain and regulate the extracellular matrix. Structurally, astrocytic fine processes enwrap synaptic contacts and exhibit remarkable plasticity resulting from crosstalk between these compartments. These features endow astrocytes with the ability to sense neuronal activity and integrate and modulate synaptic transmission, revealing them as a crucial element in mechanisms of synaptic transmission and plasticity.

Indeed, astrocyte-synapse interactions are complex and dynamic and are required for normal synaptic physiology and plasticity, as well as for the development and refinement of the neuronal circuits. Although much progress has recently been made in our understanding of the cellular and molecular mechanisms that underlie neuronal-glial interactions, ongoing research is adding new information and new questions regarding the role of glial cells in CNS development, function, and disease.

In this special issue we collected research and review articles that focus on glia-synapse interactions with a particular focus on astrocytes. An important role played by these cells in regulating long-term potentiation (LTP) and memory mechanisms in hippocampus is reviewed by Y. Ota et al. In the review “*The role of astrocytes in the regulation of synaptic plasticity and memory formation*,” the authors presented a summary of receptors and signaling molecules implicated in LTP. In addition, they propose an integrative model describing how astrocytes may modulate LTP at the postsynaptic site. Supported by a growing number of studies, their model confirms the involvement of the glutamatergic, cholinergic, and purinergic pathways in the neuron-astrocyte interactions taking place during synaptic plasticity. Moreover, this cellular interplay implicates also ephrin signaling and cytokines. Finally, Y. Ota and coauthors discuss the central role played by astrocytic calcium and associated gliotransmitters in hippocampal-dependent memory.

Although the primary function of astrocytes is to take up glutamate to prevent excitotoxicity, astrocytes are also able to release this neurotransmitter. This is generally well accepted even if the mechanisms of release remain uncertain. In this special issue, a novel mechanism of glutamate release from astrocytes was studied by C. Cali et al. in the manuscript “*G-protein coupled receptor-evoked glutamate exocytosis from astrocytes: role of prostaglandins*.” They show the role of the proinflammatory mediator prostaglandin E_2_ (PGE_2_) in glutamate exocytosis from astrocytes in the intact brain. Inhibition of cyclooxygenase pathway caused a significant reduction in the total number of fusion events of VGLUT1-positive glutamate containing vesicles in astrocytes induced by activation of purinergic and glutamatergic receptors. Prostaglandin-mediated signaling is implicated in the later, slower phase of glutamate release and requires autocrine/paracrine action of PGE_2_, suggesting a physiological role for this mediator in intercellular communication, in addition to its known role in inflammatory reactions in the brain.

Gliotransmitters glutamate and D-serine have been shown to modulate NMDA receptors (NMDAR) at extrasynaptic sites, revealing neuronal NMDAR as active components of glia to neuron communication. In the review “*GluN3A: an NMDA receptor subunit with exquisite properties and functions*,” L. A. Kehoe et al. discuss recent data on the GluN3A subunit which provides “nonconventional” properties to NMDA receptors. Expression of this subunit in early development helps to shape neuronal networks, but it may also be implicated in different neuropathologies. Interestingly, the presence of GluN3 subunit on perisynaptic astrocytic processes suggests its possible involvement in neuron-glia interactions, although more research is required to elucidate this question.

Another mechanism used by astrocytes to regulate intercellular interactions in the CNS is through secretion of matricellular proteins. These proteins are nonstructural molecules that regulate the extracellular matrix and cell-cell interactions. In the paper “*Astrocyte-secreted matricellular proteins in CNS remodelling during development and disease*,” E. V. Jones and D. S. Bouvier review the roles of matricellular proteins secreted from developing and reactive astrocytes in CNS development, injury, and disease and discuss their potential as therapeutic targets.

In the paper “*Astrocyte-synapse structural plasticity*,” Y. Bernardinelli et al. review the data on plasticity of perisynaptic astrocytic processes (PAPs). The authors discuss electron and optical microscopy data showing the distribution of fine astrocytic processes around synapses and overview growing evidence on PAP structural plasticity. Although the exact mechanisms and roles of this type of astrocytic plasticity are still not clear, recent data has revealed a requirement for neuronal activity and suggests that PAPs may be implicated in the structural support and plasticity of a synapse, control of neurotransmission and intersynaptic crosstalk, energy supply, maintenance of extracellular homeostasis, and integration of synaptic signals.

The study of glial structural plasticity (such as PAPs) requires precise quantification of fine processes and their motility. This challenging task is addressed in the paper “*Improved method for the quantification of motility in glia and other morphologically complex cells*” by M. Sild et al. who propose and describe in detail a new approach to calculate a motility index for cells with complex, dynamic morphologies.

In this special issue, only some aspects out of a broad range of topics on synapse-glia interactions are highlighted and discussed. Despite great progress made recently in our understanding of glia and their role in the CNS, there is still a long road ahead. We hope that the data presented in this issue will help the future research in this quickly growing and important field.

